# Cell entry of bovine respiratory syncytial virus through clathrin-mediated endocytosis is regulated by PI3K-Akt and Src-JNK pathways

**DOI:** 10.3389/fmicb.2024.1393127

**Published:** 2024-04-16

**Authors:** Yang Liu, Dongliang Yang, Wen Jiang, Tianying Chi, Jingli Kang, Zhiliang Wang, Faxing Wu

**Affiliations:** ^1^Key Laboratory of Animal Biosafety Risk Prevention and Control of Ministry of Agriculture and Rural Affairs (South), China Animal Health and Epidemiology Center, Qingdao, Shandong, China; ^2^Key Laboratory of Biotechnology and Bioengineering of State Ethnic Affairs Commission, Biomedical Research Center, Northwest Minzu University, Lanzhou, Gansu, China

**Keywords:** bovine respiratory syncytial virus, endocytosis, clathrin, dynamin, endosomes

## Abstract

Bovine respiratory syncytial virus (BRSV) is an RNA virus with envelope that causes acute, febrile, and highly infectious respiratory diseases in cattle. However, the manner and mechanism of BRSV entry into cells remain unclear. In this study, we aimed to explore the entry manner of BRSV into MDBK cells and its regulatory mechanism. Our findings, based on virus titer, virus copies, western blot and IFA analysis, indicate that BRSV enters MDBK cells through endocytosis, relying on dynamin, specifically via clathrin-mediated endocytosis rather than caveolin-mediated endocytosis and micropinocytosis. We observed that the entered BRSV initially localizes in early endosomes and subsequently localizes in late endosomes. Additionally, our results of western blot, virus titer and virus copies demonstrate that BRSV entry through clathrin-mediated endocytosis is regulated by PI3K-Akt and Src-JNK signaling pathways. Overall, our study suggests that BRSV enters MDBK cells through clathrin-mediated endocytosis, entered BRSV is trafficked to late endosome via early endosome, BRSV entry through clathrin-mediated endocytosis is regulated by PI3K-Akt and Src-JNK signaling pathways.

## 1 Introduction

Bovine respiratory syncytial virus (BRSV) is an RNA virus with envelope, belongs to the paramyxovirus family. It is highly infectious and can be spread through aerosols and direct contact between animals (Brodersen, 2010). The incubation period of BRSV in cattle is typically 2 to 5 days, during which it replicates in the cells of the respiratory tract and lungs. Following the incubation period, infected cattle exhibit clinical symptoms such as fever, cough, and increased nasal and ocular discharge (Larsen, 2000; Valarcher and Taylor, 2007). As a result, this disease causes significant economic losses in the cattle industry (Santos-Rivera et al., 2022).

Virus entry into cells is an important step in their life cycle, virus completes its own replication and proliferation in the cells after entry (Yamauchi and Helenius, 2013). The common method for viruses to enter cells is through endocytosis, which can be categorized into three types based on the proteins involved: clathrin-mediated endocytosis, caveolin-mediated endocytosis and macropinocytosis (Mercer et al., 2010). Caveolin-mediated endocytosis relies on caveolae, which are structures on the cell membrane containing cholesterol, and dynamin plays a significant role in this process (Shikanai et al., 2018; Xia et al., 2018). Clathrin-mediated endocytosis, which also depends on dynamin, is another common endocytosis for virus entry (Sun and Tien, 2013). Macropinocytosis depends on membrane ruffles enveloping the virus-receptor complex and promoting virus internalization (Mercer et al., 2010; Han et al., 2016).

During the early stage of infection, the virus binds to the cell receptor and initiates downstream signaling pathways to facilitate its entry into cells (Ewers and Helenius, 2011). Previous studies have reported that PI3K-Akt signaling pathway can regulate virus replication or entry, such as herpes simplex virus 1 (HSV-1), Bovine ephemeral fever virus (BEFV) and Ebola virus (EBoV; Li et al., 2010; Feng et al., 2011; Cheshenko et al., 2013; Cheng et al., 2015). Furthermore, the Src-JNK signaling pathway has been found to be activated during BEFV infection, promoting clathrin-dependent endocytosis and facilitating BEFV entry into cells (Cheng et al., 2015). However, the specific regulatory mechanism by which BRSV enters the cell remains poorly understood.

In this study, we investigated the route and mechanism of BRSV entry into MDBK cells. Our results indicate that BRSV enters MDBK cells through clathrin-mediated endocytosis rather than caveolin-mediated endocytosis and macropinocytosis, internalized BRSV is sequentially located in early endosome and late endosome. The entry process of BRSV is regulated by PI3K-Akt and Src-JNK signaling pathways.

## 2 Materials and methods

### 2.1 Cells and viruses

MDBK cells were provided by China Animal Health and Epidemiology Center, it was cultured with DMEM (Procell Life Science, Wuhan, China) contains 10% FBS (Jinyuankang Bio-engineering, Guangzhou, China). BRSV Yunnan strain isolated and characterized in 2021 was provided by China Animal Health and Epidemiology Center.

### 2.2 Reagents and antibodies

Wortmannin, dynasore, SU6655, NH_4_Cl, SP600125, IPA-3, MβCD and chlorpromazine were obtained from Sigma (Sigma, MO, United States), Akti-1/2 and bafilomycin A1 were obtained from Abcam (Abcam, Cambridge, United Kingdom), nystatin and EIPA was obtained from Solarbio (Solarbio, Beijing, China).

The rabbit anti-dynamin-2, anti-clathrin heavy chain, anti-Rab5, anti-Rab7, anti-PI3K p85 (phos pho Y458) + PI3 Kinase p55 (phospho Y199), anti-Akt, anti-Src, anti-Src (phospho Y416), anti-phospho-Akt (Ser473) and anti-GAPDH monoclonal antibodies were obtained from Abcam (Abcam, Cambridge, United Kingdom). The rabbit anti-JNK and anti-JNK (Thr183/Tyr185) monoclonal antibodies were obtained from Cell Signaling Technology (Cell Signaling Technology, Danvers, United States). The rabbit anti-PI3 Kinase p85 alpha polyclonal antibody, the mouse anti-Flag monoclonal antibody, goat anti-rabbit and goat anti-mouse HRP-labeled secondary antibody, Alexa fuor-488-conjugated anti-mouse, Cy™3-conjugated anti-rabbit IgG (H + L) and mounting medium with DAPI was purchased from Abcam (Abcam, Cambridge, United Kingdom). The rabbit anti-caveolin-1 monoclonal antibody was obtained from Beyotime (Beyotime Biotechnology, Shanghai, China). The mouse anti-BRSV G protein monoclonal antibody was provided by China Animal Health and Epidemiology Center.

### 2.3 Cell viability determination

MDBK cells were inoculated onto a 96-well plate and treated with various inhibitors for 24 h after reaching full confluence, the initial adding dose of various inhibitors was referred to previous reports (Cheng et al., 2012; Yang et al., 2018; Hou et al., 2022; Liu et al., 2023). Subsequently, CCK-8 reagent (Vazyme Biotech, Nanjing, China) was added. Following incubation at 37°C for 4 h, the absorbance was measured at 450 nm using the microplate reader (Flash Spectrum Biotechnology, Shanghai, China).

### 2.4 Viral entry and replication

Inhibitor was added 1 h before or after BRSV infecting MDBK cells at a multiplicity of infection (MOI) of 2, the cells were incubated at 4°C for 1 h to allow the virus to adsorb on the cell surface, the cells were washed with PBS repeatedly to remove the unabsorbed BRSV particles, and cultivated in DMEM with 2% FBS at 37°C for 1 or 24 h in the presence of inhibitor. Supernatant from cells cultivated for 24 h was collected to detect virus viral entry and replication by TCID_50_. Additionally, cells infected for 1 h were treated with proteinase K at 4°C to remove the virus that had not entered, followed by treatment with 2 mM phenylmethylsulfonyl fluoride (PMSF). RNA was then extracted to detect virus entry using qPCR.

### 2.5 qPCR

Cells were collected and RNA was extracted for reverse transcription. The real-time PCR system (TIANLONG, Xian, China) was used to detect Ct, using primer BRSV-N qF: TGAAAAGYACCCTCATTACAT, BRSV-N qR: CATCACTTGACCTGCTCCAT and probe: TGCAGGGTTATTCATGAATGCATATGGA, the BRSV copies were calculated as follows: 10^(47.686-Ct)/3.466^.

### 2.6 TCID_50_ analysis

MDBK cells were inoculated on 96-well plate 24 h before the experiment. Once the cells reached full confluence, the virus was serially diluted 10 times and then inoculated into the MDBK cells. The infected cells were incubated at 37°C for 1 h. Subsequently, the cells were repeatedly washed by PBS and supplemented with 2% FBS DMEM. The wells exhibiting cytopathic effects (CPE) were counted after 5 to 7 days of culturing at 37°C, and TCID_50_ was determined by Reed-Muench formula.

### 2.7 Immunofluorescence

The infected cells were fixed at 4°C with 70% ethanol for 1 h, washed three times with PBS, and incubated at 37°C for 1 h with specific antibody and washed again with PBS. The cells were then incubated with DAPI at 37°C for 10 min, washed three times with pure water, fixed with mounting medium, and analyzed with the confocal microscope Leica STELLARIS 5 (Leica, Frankfurt, Germany).

### 2.8 siRNA transfections

The siRNA was conducted in this study was designed and synthesized by Tsingke Biotechnology (Tsingke Biotechnology, Beijing, China), the sequences of siRNA are shown in Table 1. MDBK cells were inoculated on 6-well plate, siRNA was transfected into MDBK cells using Lipofectamine™ 3,000 (TermoFisher, MA, United States) according to the instruction once the cell confluence reached 70% to 80%, and the effect of protein silencing was assessed by western blot.

**Table 1:** The sequences of the siRNA.

siRNA name	Sequence (5′-3′)
Dynamin2-siRNA1	GAGCTAATCAATACAGTTA
Dynamin2-siRNA2	GCTACATTGGAGTGGTCAA
Dynamin2-siRNA3	CGAAGGCCTTCATCCACTA
CHC-siRNA1	GCTAAGTTGTTGTACAATA
CHC-siRNA2	CGATAGATGCTTATGACAA
CHC-siRNA3	CGAGGTTGCTTGAGATGAA
Cav1-siRNA1	CTGCACATCTGGGCAGTTG
Cav1-siRNA2	CCACCTTCACTGTGACAAA
Cav1-siRNA3	CGACGACGTGGTCAAGATT
Rab5-siRNA1	CACAAGACAGGCCAAGAAA
Rab5-siRNA2	ACCCGCAAGGTTACCACAG
Rab5-siRNA3	CATCACCAAGTGCAGCAAG
Rab7-siRNA1	TTCAGCAAACACTACAAGT
Rab7-siRNA2	TTCCCAGGACAGCTTCAGC
Rab7-siRNA3	GGTTTCACAGGTTGGACAG

### 2.9 Western blot

The cell lysate samples were treated with high temperature, electrophoresed on a 12% SDS-PAGE gel, and then transferred to a polyvinylidene fluoride (PVDF) membrane. The PVDF membrane was incubated with 5% milk at room temperature for 1 h, followed by incubated with primary antibody at 4°C overnight, and incubated with secondary antibody at room temperature for 1 h. The bands on the PVDF membrane were analyzed by ECL chemiluminescence reagent (Beyotime Biotechnology, Shanghai, China) and chemiluminescence analyzer (UVITEC, Cambridge, England).

### 2.10 Statistical analyses

The experimental results were obtained from three sets of independent experiments. One-way ANOVA analysis was conducted on the data using Graphpad Prism Version 5.0. The significance of differences was indicated by asterisks as follows: ^*^p < 0.05, ^**^p < 0.01, and ^***^p < 0.001.

## 3 Results

### 3.1 BRSV entry is dependent on low pH

**Figure 1:**
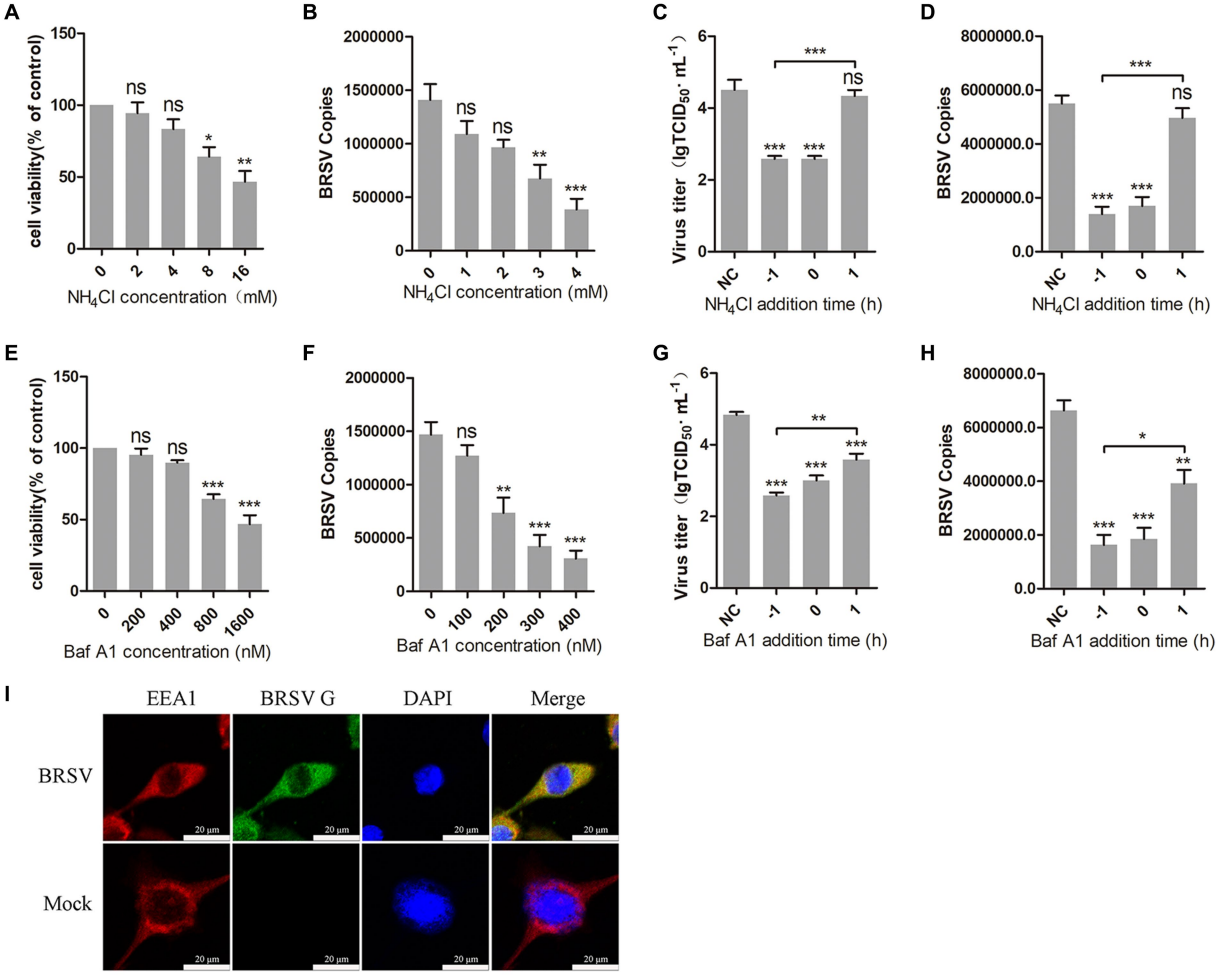
BRSV entry into MDBK cells requires low pH. **(A,E)** MDBK cells treated with different concentrations of NH_4_Cl or bafilomycin A1 (Baf A1) were analyzed by CCK-8 reagent to test cell viability. **(B,F)** MDBK cells were pretreated with NH_4_Cl or Baf A1 for 1 h, bound with BRSV at a multiplicity of infection (MOI) of 2, transferred to 4°C for 1 h, and then transferred to 37°C for 1 h. The infected cells were collected, BRSV entry was analyzed by virus copies. **(C,G)** NH_4_Cl or Baf A1 was added 1 h before or after BRSV infecting MDBK cells, the cells bound with BRSV at 4°C for 1 h were transferred to 37°C for 24 h. BRSV entry and replication were analyzed by virus titer, **(D,H)** BRSV entry and replication were analyzed by virus copies. **(I)** MDBK cells were infected with BRSV at an MOI of 5 and incubated for 1 h at 4°C, incubated for 30 min at 37°C. Confocal microscope analysis of EEA1 (red), BRSV G protein (green) and cell nucleus (blue) in BRSV entering MDBK cells. Scale bar = 20 μm. ^*^p < 0.05; ^**^p < 0.01; ^***^p < 0.001.

The internalization of most enveloped viruses relies on the acidic endosomal environment (Más and Melero, 2013; Nicola, 2016). To examine whether BRSV entry into MDBK cells is dependent on a low pH, we examined the effect of two endosome acidification inhibitors, NH_4_Cl and bafilomycin A1, on BRSV entry. Different concentrations of NH_4_Cl and bafilomycin A1 were added to MDBK cells and the activity of MDBK cells was examined by MTT assay. NH_4_Cl greater than 4 mM and bafilomycin A1 greater than 400 nM decreased the viability of MDBK cells (Figures 1A,E). MDBK cells pretreated with NH_4_Cl and bafilomycin A1 at 37°C for 1 h were incubated with BRSV at 4°C for 1 h, so that the virus was adsorbed on the cells surface in the presence of inhibitors, the unbound virus was removed, and the virus entry was assessed after incubation at 37°C for 1 h. The results of virus copies demonstrated that both NH_4_Cl and bafilomycin A1 inhibited BRSV entry into MDBK cells in a dose-dependent manner (Figures 1B,F). Subsequently, we investigated the role of low pH in BRSV entry and replication by adding NH_4_Cl and bafilomycin A1 to MDBK cells 1 h before and after BRSV infection, the cells inoculated with BRSV only were used as controls. The results of viral titer and viral copies showed that NH_4_Cl only inhibited the entry process of BRSV (Figures 1C,D), whereas bafilomycin A1 inhibited both entry and replication of BRSV (Figures 1G,H). Endocytosis is dependent on endosomes, and EEA1 is the hallmark protein of early endosomes (Mills et al., 1998; Ramanathan and Ye, 2012). To further demonstrate the role of endocytosis in BRSV entry, we examined the localization of BRSV and EEA1 in the early stage of BRSV infecting MDBK cells. MDBK cells incubated with BRSV at 4°C for 1 h were transferred to 37°C for 30 min, the localization of EEA1 and BRSV G protein was analyzed by IFA and confocal microscopy, the result showed clear co-localization of EEA1 and BRSV G protein after 30 min of infection (Figure 1I). In conclusion, these results suggest that the acidic endosomal environment plays a significant role in BRSV entry.

### 3.2 BRSV entry is dependent on dynamin

**Figure 2:**
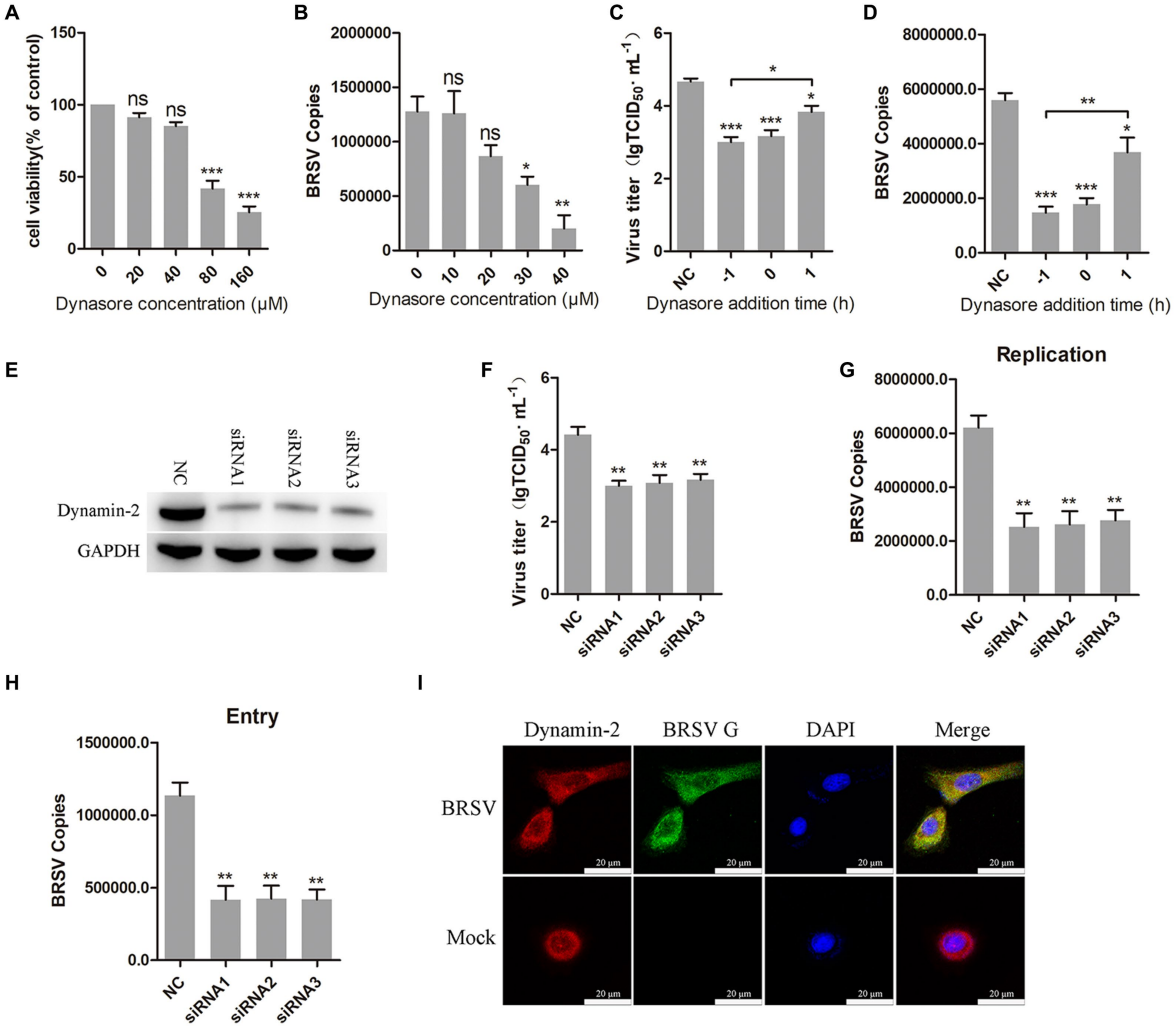
BRSV entry into MDBK cells requires dynamin. **(A)** MDBK cells treated with different concentrations of dynasore were analyzed by CCK-8 reagent to test cell viability. **(B)** MDBK cells were pretreated with dynasore for 1 h, bound with BRSV at 4°C for 1 h, and transferred to 37°C for 1 h. The infected cells were collected, BRSV entry was analyzed by virus copies. **(C)** Dynasore was added 1 h before or after BRSV infecting MDBK cells, the cells bound with BRSV at 4°C for 1 h were transferred to 37°C for 24 h. BRSV entry and replication were analyzed by virus titer, **(D)** BRSV entry and replication were analyzed by virus copies. **(E)** Dynamin-2 siRNA was transfected into MDBK cells, the silencing effect was detected by western blot after 48 h. **(F)** Dynamin-2 siRNA was transfected into MDBK cells, the cells were infected with BRSV at an MOI of 2 for 24 h, BRSV replication was detected by virus titer, **(G)** BRSV replication was detected by virus copies. **(H)** Dynamin-2 siRNA was transfected into MDBK cells, the cells were infected with BRSV at an MOI of 2 for 1 h, BRSV entry was detected by virus copies. **(I)** MDBK cells were infected with BRSV at an MOI of 5 and incubated for 1 h at 4°C, incubated for 30 min at 37°C. Confocal microscope analysis of dynamin-2 (red), BRSV G protein (green) and cell nucleus (blue) in BRSV entering MDBK cells. Scale bar = 20 μm. ^*^p < 0.05; ^**^p < 0.01; ^***^p < 0.001.

Dynamin is a large GTPase involved in the separation of vesicles from cell membranes during endocytosis, it plays an important role in clathrin-mediated and caveolin-mediated endocytosis (Sun and Tien, 2013). To investigate the role of dynamin in BRSV entry, we used dynasore, a dynamin inhibitor (Macia et al., 2006). Different concentrations of dynasore were added to MDBK cells and the activity of MDBK cells was examined by MTT assay. We observed that dynasore greater than 40 μM reduces MDBK cell viability (Figure 2A). MDBK cells pretreated with dynasore at 37°C for 1 h were incubated with BRSV at 4°C for 1 h and transferred to 37°C for 1 h. The result of viral copies showed that dynasore inhibited BRSV entry into MDBK cells in a dose-dependent manner (Figure 2B). To further examine the role of dynamin in BRSV entry and replication, we added dynasore to MDBK cells 1 h before and after BRSV infection, the cells inoculated with BRSV only were used as controls. Virus titer and virus copies demonstrated that dynasore inhibited BRSV entry and replication (Figures 2C,D). We also focused on dynamin-2, a vital protein of dynamin family. Small interfering RNA (siRNA) of dynamin-2 was transfected into MDBK cells, and the silencing effect was detected by western blot after 48 h. The result revealed the expression of dynamin-2 was significantly silenced (Figure 2E). we next infected the cells with BRSV for 24 and 1 h to assess the effects of dynamin-2 on BRSV replication and entry. The measurements of viral titer and viral copies revealed that silencing dynamin-2 inhibited the replication of BRSV in MDBK cells (Figures 2F,G) as well as entry (Figure 2H). Additionally, MDBK cells incubated with BRSV at 4°C for 1 h were transferred to 37°C for 30 min, the localization of dynamin-2 and BRSV G protein was analyzed by IFA and confocal microscopy, the result showed clear co-localization of dynamin-2 and G protein after 30 min of infection (Figure 2I). These results indicate that dynamin plays a crucial role in BRSV entry.

### 3.3 BRSV entry is dependent on clathrin-mediated endocytosis

**Figure 3:**
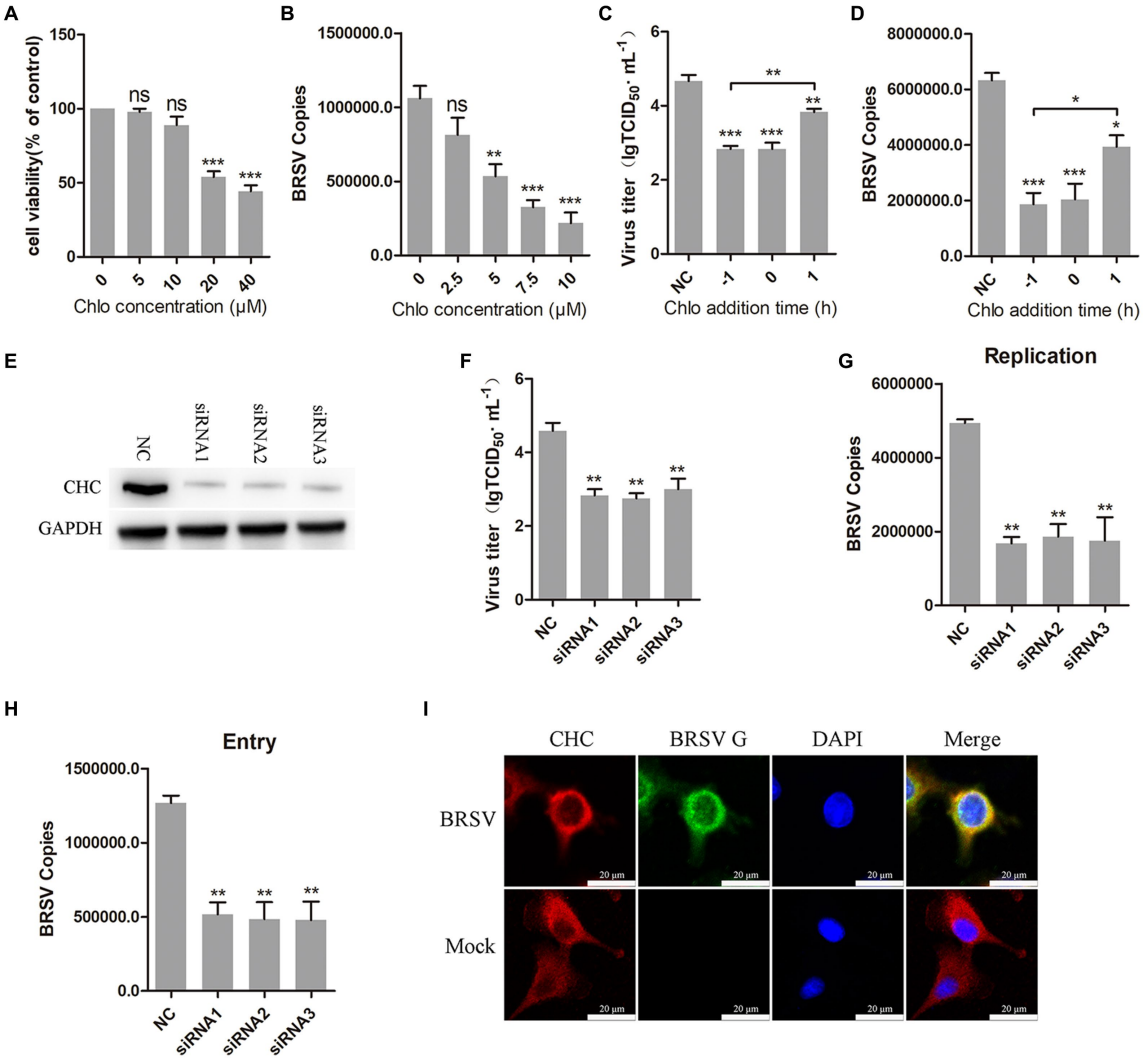
BRSV entry into MDBK cells requires clathrin-mediated endocytosis. **(A)** MDBK cells treated with different concentrations of chlorpromazine (Chlo) were analyzed by CCK-8 reagent to test cell viability. **(B)** MDBK cells were pretreated with Chlo for 1 h, bound with BRSV at 4°C for 1 h, and transferred to 37°C for 1 h. The infected cells were collected, BRSV entry was analyzed by virus copies. **(C)** Chlo was added 1 h before or after BRSV infecting MDBK cells, the cells bound with BRSV at 4°C for 1 h were transferred to 37°C for 24 h. BRSV entry and replication were analyzed by virus titer, **(D)** BRSV entry and replication were analyzed by virus copies. **(E)** Clathrin heavy chain protein (CHC) siRNA was transfected into MDBK cells, the silencing effect was detected by western blot after 48 h. **(F)** CHC siRNA was transfected into MDBK cells, the cells were infected with BRSV at an MOI of 2 for 24 h, BRSV replication was detected by virus titer, **(G)** BRSV replication was detected by virus copies. **(H)** CHC siRNA was transfected into MDBK cells, the cells were infected with BRSV at an MOI of 2 for 1 h, BRSV entry was detected by virus copies. **(I)** MDBK cells were infected with BRSV at an MOI of 5 and incubated for 1 h at 4°C, incubated for 30 min at 37°C. Confocal microscope analysis of CHC (red), BRSV G protein (green) and cell nucleus (blue) in BRSV entering MDBK cells. Scale bar = 20 μm. ^*^p < 0.05; ^**^p < 0.01; ^***^p < 0.001.

Clathrin-mediated endocytosis is a dynamin-dependent endocytosis (Sun and Tien, 2013). To investigate the involvement of clathrin-mediated endocytosis in BRSV entry, we selected chlorpromazine, an inhibitor that prevents the aggregation and formation of clathrin-coated pits on the cell surface. Treatment with chlorpromazine at concentrations greater than 10 μM led to a decrease in MDBK cell viability (Figure 3A). MDBK cells pretreated at 37°C with chlorpromazine for 1 h were incubated with BRSV at 4°C for 1 h and transferred to 37°C for 1 h, the results of qPCR showed that BRSV entry was significantly inhibited (Figure 3B). The role of clathrin-mediated endocytosis in BRSV entry and replication was detected by adding chlorpromazine to MDBK cells 1 h before and after BRSV inoculation, the cells inoculated with BRSV only were used as controls. The results of virus titer and virus copies indicated that chlorpromazine inhibited BRSV entry and replication (Figures 3C,D). Clathrin heavy chain protein (CHC) is an important functional domain of clathrin (Li et al., 2010). To further determine the role of clathrin-mediated endocytosis in BRSV entry, small interfering RNA (siRNA) of CHC was transfected into MDBK cells, and the silencing effect was detected by western blot after 48 h. The result revealed the expression of CHC was significantly silenced (Figure 3E). We inhibited CHC expression in MDBK cells by siRNA of CHC, and then assessed virus replication after inoculation with BRSV for 24 h. Virus titer and virus copies showed that silencing CHC inhibited virus replication (Figures 3F,G). After silencing CHC, we inoculated BRSV for 1 h and detected the virus entry, the result of virus copies revealed that silencing CHC inhibited the entry of BRSV (Figure 3H). Furthermore, MDBK cells incubated with BRSV at 4°C for 1 h were transferred to 37°C for 30 min, and the localization of CHC and BRSV G protein was analyzed by IFA and confocal microscopy. The result showed clear co-localization of CHC and G protein after 30 min of infection (Figure 3I). These results indicate that BRSV entry into MDBK cells depends on clathrin-mediated endocytosis.

### 3.4 BRSV entry is independent on caveolin-mediated endocytosis

**Figure 4:**
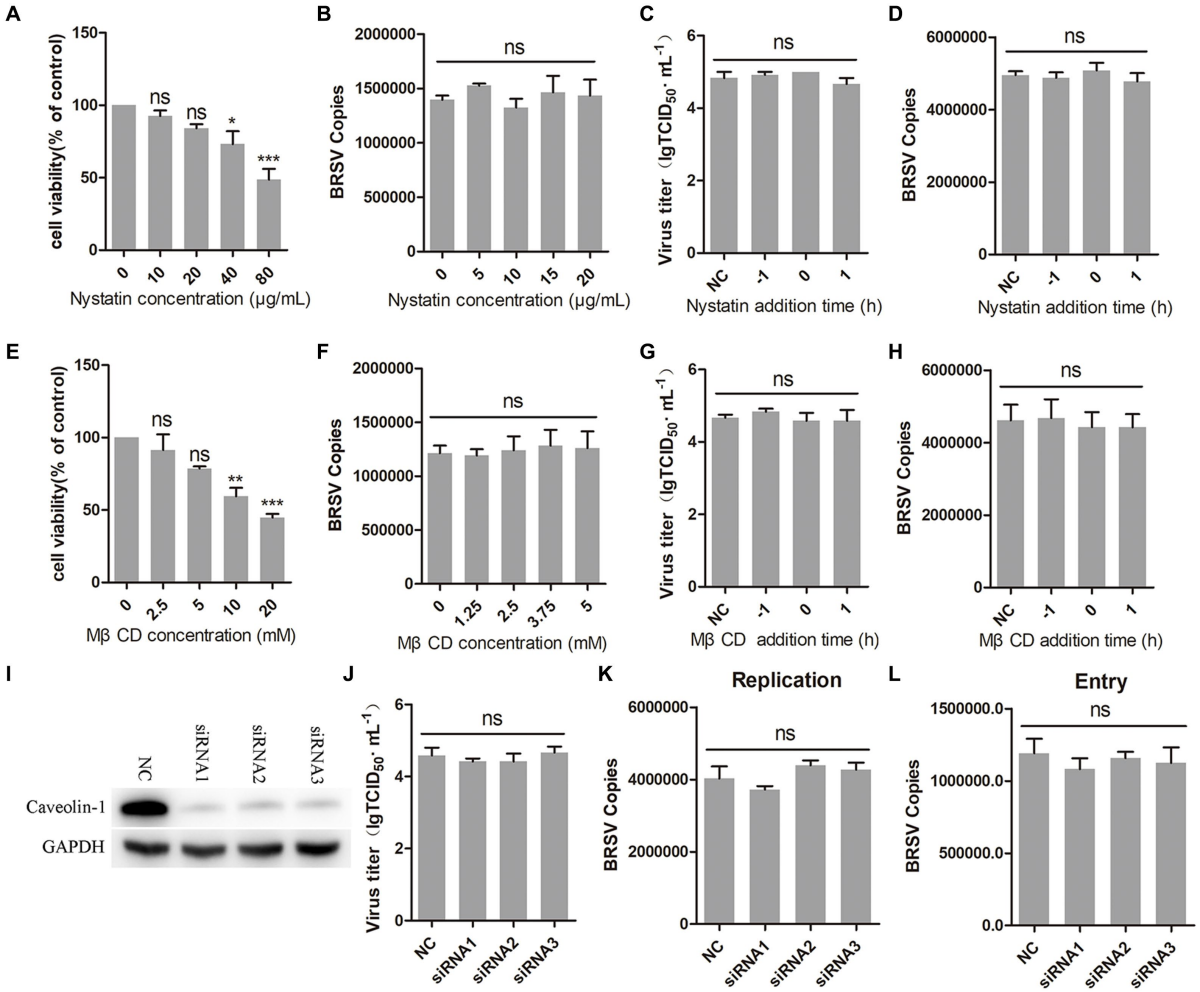
BRSV entry does not require caveolin-mediated endocytosis. **(A,E)** MDBK cells treated with different concentrations of nystatin or MβCD were analyzed by CCK-8 reagent to test cell viability. **(B,F)** MDBK cells were pretreated with nystatin or MβCD for 1 h, bound with BRSV at 4°C for 1 h, and transferred to 37°C for 1 h. The infected cells were collected, BRSV entry was analyzed by virus copies. **(C,G)** Nystatin or MβCD was added 1 h before or after BRSV infecting MDBK cells, the cells bound with BRSV at 4°C for 1 h were transferred to 37°C for 24 h. BRSV entry and replication were analyzed by virus titer, **(D,H)** BRSV entry and replication were analyzed by virus copies. **(I)** Caveolin-1 siRNA was transfected into MDBK cells, the silencing effect was detected by western blot after 48 h. **(J)** Caveolin-1 siRNA was transfected into MDBK cells, the cells were infected with BRSV at an MOI of 2 for 24 h, BRSV replication was detected by virus titer, **(K)** BRSV replication was detected by virus copies. **(L)** Caveolin-1 siRNA was transfected into MDBK cells, the cells were infected with BRSV at an MOI of 2 for 1 h, BRSV entry was detected by virus copies. ^*^p < 0.05; ^**^p < 0.01; ^***^p < 0.001.

Caveolin-mediated endocytosis is a type of endocytosis that involves dynamin (Orth et al., 2002). We investigated whether caveolin-mediated endocytosis plays a role in BRSV entry by using two inhibitors, nystatin and MβCD, which are known to block this process. Caveolin-mediated endocytosis relies on the uptake capacity of caveolae, which are structures that require cholesterol on the cell membrane (Shikanai et al., 2018; Xia et al., 2018). Nystatin and MβCD can deplete cholesterol on cell membranes, thereby inhibiting caveolin-mediated endocytosis. We detected the appropriate working concentrations of nystatin and MβCD, the results of cell viability detection showed that nystatin greater than 20 μg/mL and MβCD greater than 5 mM significantly inhibited the cell viability of MDBK cells (Figures 4A,E). MDBK cells pretreated at 37°C with nystatin or MβCD for 1 h were incubated with BRSV at 4°C for 1 h and transferred to 37°C for 1 h, our results of virus copies showed that nystatin and MβCD did not affect the entry of BRSV into MDBK cells (Figures 4B,F). We added nystatin and MβCD to MDBK cells 1 h before and after BRSV inoculation to analyze the role of caveolin-mediated endocytosis in BRSV entry and replication, the cells inoculated with BRSV only were used as controls. Virus titer detection revealed that nystatin and MβCD did not affect BRSV entry and replication (Figures 4C,G), which was also supported by the results of virus copies detection (Figures 4D,H). Caveolin-1 is a crucial protein in the caveolin family, plays a significant role in caveolae formation and stability (Pelkmans and Helenius, 2002), so we used the siRNA to silence caveolin-1 (Figure 4I), and then inoculated BRSV for 24 h to detect BRSV replication. The results of virus titer and virus copies showed that silencing caveolin-1 did not affect the replication of BRSV (Figures 4J,K). We inoculated BRSV for 1 h after silencing caveolin-1 of MDBK cells, virus copies analysis showed that silencing caveolin-1 did not affect BRSV entry (Figure 4L). These findings suggest that BRSV entry into MDBK cells is independent on caveolin-mediated endocytosis.

### 3.5 BRSV entry is independent on macropinocytosis

**Figure 5:**
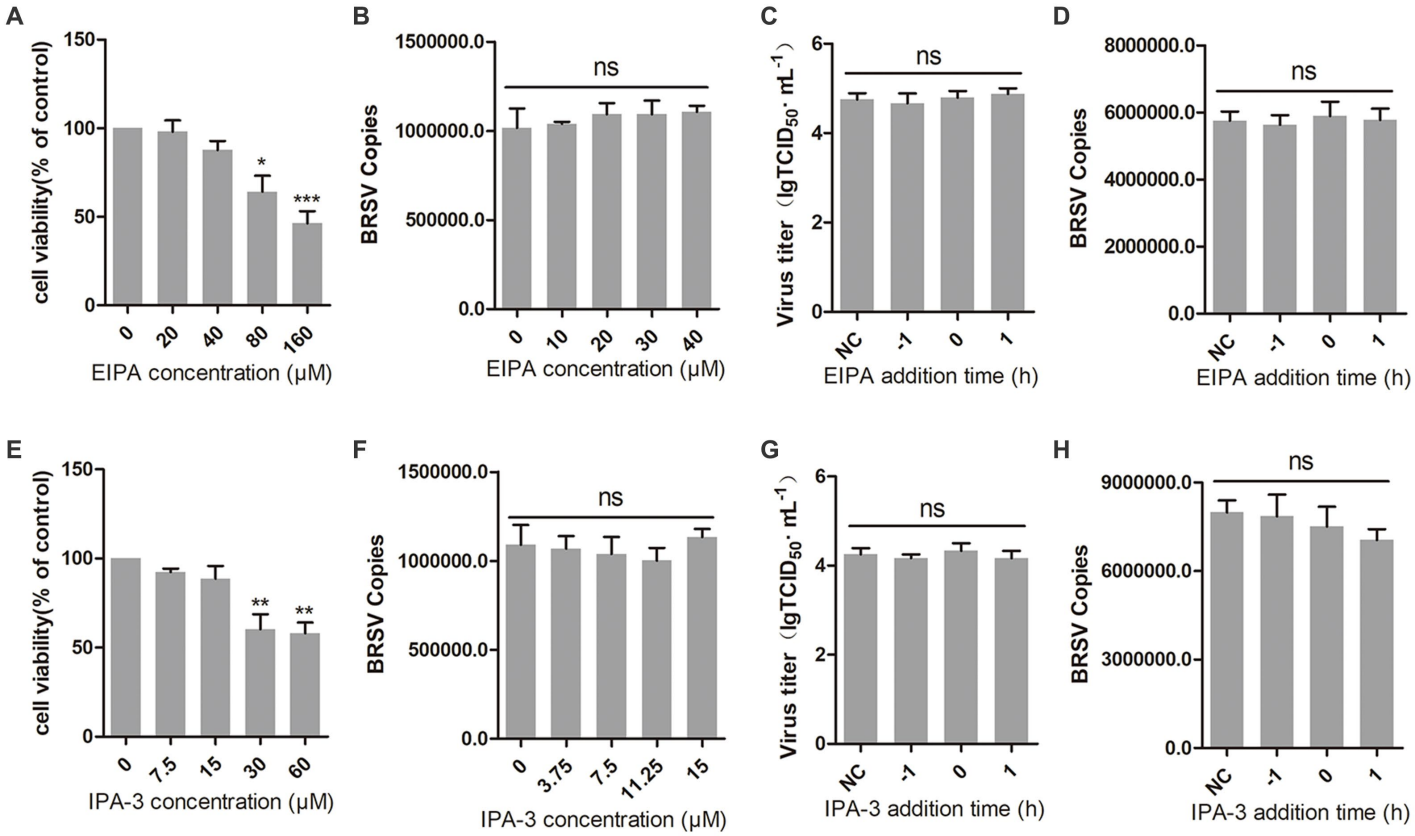
BRSV entry does not require macropinocytosis. **(A,E)** MDBK cells treated with different concentrations of EIPA or IPA-3 were analyzed by CCK-8 reagent to test cell viability. **(B,F)** MDBK cells were pretreated with EIPA or IPA-3 for 1 h, bound with BRSV at 4°C for 1 h, and transferred to 37°C for 1 h. The infected cells were collected, BRSV entry was analyzed by virus copies. **(C,G)** EIPA or IPA-3 was added 1 h before or after BRSV infecting MDBK cells, the cells bound with BRSV at 4°C for 1 h were transferred to 37°C for 24 h. BRSV entry and replication were analyzed by virus titer, **(D,H)** BRSV entry and replication were analyzed by virus copies. ^*^p < 0.05; ^**^p < 0.01; ^***^p < 0.001.

Macropinocytosis is also a common manner of viral entry into cells (Coyne et al., 2007; Krieger et al., 2013). During virus entry through macropinocytosis, cell membrane forms ruffles that envelop the virus-receptor complex and create macropinosomes, which transport the virus into the cell (Mercer et al., 2010; Han et al., 2016). This process relies on the function of Na^+^/H^+^ exchangers (NHE). EIPA, an NHE inhibitor, was used to inhibit the macropinocytosis of MDBK cells (Koivusalo et al., 2010). The result of cell viability showed that EIPA greater than 40 μM inhibited the viability of MDBK cells (Figure 5A). We inoculated MDBK cells with EIPA and subsequently inoculated them with BRSV to assess BRSV entry, the detection result of virus copies showed that EIPA had no effect on BRSV entry into MDBK cells (Figure 5B). To examine the role of macropinocytosis in BRSV entry and replication, we added EIPA to MDBK cells 1 h before and after BRSV infection, the cells inoculated with BRSV only were used as controls. The results of virus titer and virus copies showed that EIPA did not affect BRSV entry and replication (Figures 5C,D). During virus entry via macropinocytosis, the formation of membrane ruffles depends on actin rearrangement, p21-activated kinase 1 (Pak1) is a member of the PAK family and promotes actin rearrangement (Han et al., 2016). To further determine the dependence of BRSV entry into MDBK cells on macropinocytosis, we treated MDBK cells with the Pak1 inhibitor, IPA-3 (Li et al., 2021). The result of cell viability showed that IPA-3 greater than 15 μM inhibited the viability of MDBK cells (Figure 5E). MDBK cells were treated with IPA-3 and then inoculated with BRSV to detect virus entry, the results of virus copies showed that IPA-3 had no influence on BRSV entry (Figure 5F). We also examined the role of macropinocytosis in BRSV entry and replication by adding IPA-3 to MDBK cells 1 h before and after BRSV infection, the results of virus titer and virus copies showed that IPA-3 did not affect BRSV entry and replication (Figures 5G,H). These results suggest that BRSV entry into MDBK cells is independent on macropinocytosis.

### 3.6 Entered BRSV is trafficked to late endosomes via early endosomes

**Figure 6:**
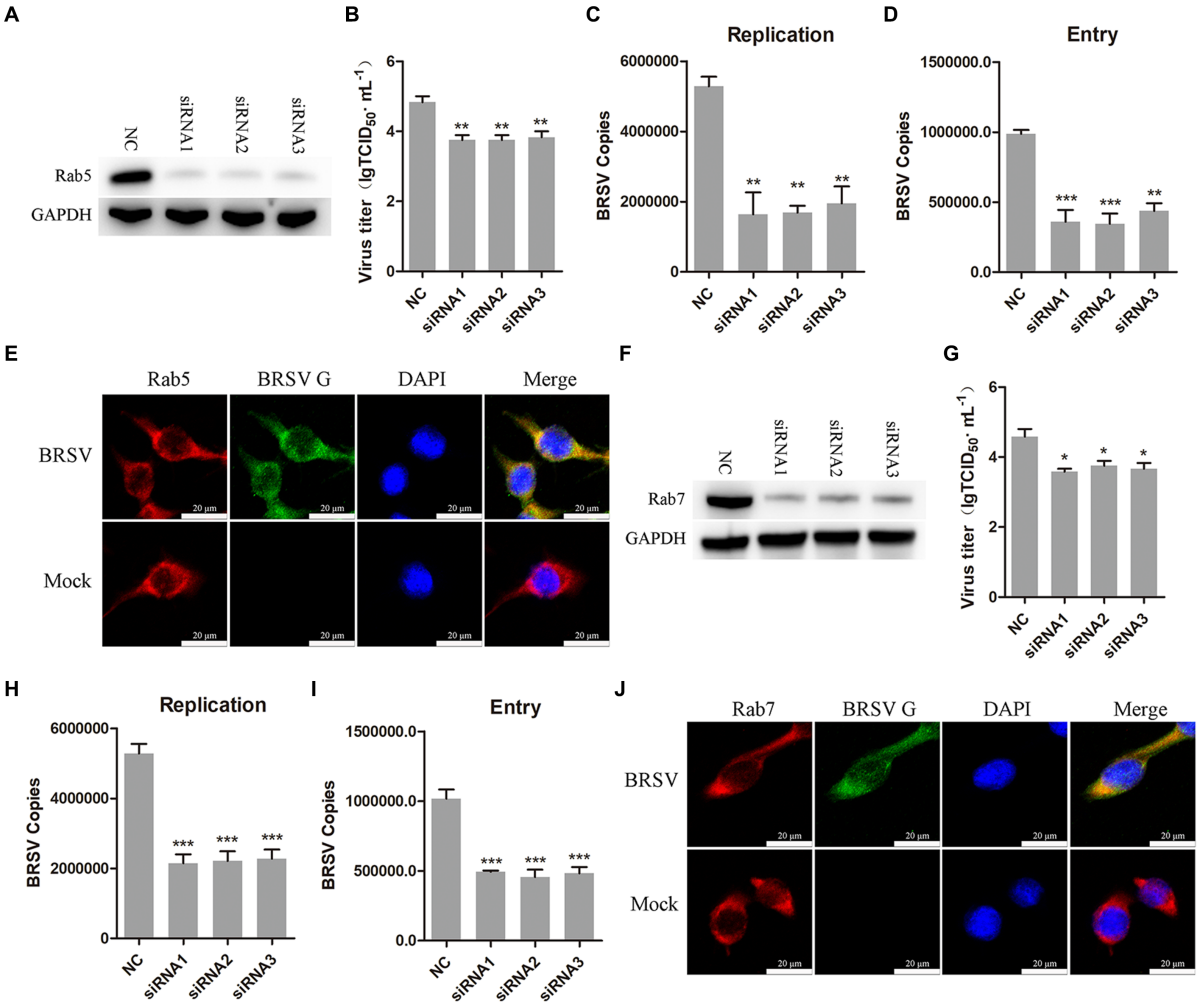
Internalized BRSV is located in the early endosome and late endosome successively. **(A,F)** The siRNA of Rab5 or Rab7 was transfected into MDBK cells, the silencing effect was detected by western blot after 48 h. **(B,G)** The siRNA of Rab5 or Rab7 was transfected into MDBK cells, the cells were infected with BRSV at an MOI of 2 for 24 h, BRSV replication was detected by virus titer, **(C,H)** BRSV replication was detected by virus copies. **(D,I)** The siRNA of Rab5 or Rab7 was transfected into MDBK cells, the cells were infected with BRSV at an MOI of 2 for 1 h, BRSV entry was detected by virus copies. **(E,J)** MDBK cells were infected with BRSV at an MOI of 5 and incubated for 1 h at 4°C, incubated for 30 min/60 min at 37°C, confocal microscope analysis of Rab5/Rab7 (red), BRSV G protein (green) and cell nucleus (blue) in BRSV entering MDBK cells. Scale bar = 20 μm. ^*^p < 0.05; ^**^p < 0.01; ^***^p < 0.001.

After internalization, early endosomes mature into late endosomes by increasing intracavitary acidity. Late endosomes can fuse with each other to form larger vesicles, primarily existing as the multivesicular body (MVB; Hanson and Cashikar, 2012). Rab proteins play a role in the membrane transport during this process (Vale-Costa and Amorim, 2016). Early endosomes are rich in Rab5, late endosomes release Rab5, incorporate Rab7, and fuse with lysosomes (Rink et al., 2005). Our previous results have demonstrated that hallmark protein EEA1 of early endosome and the BRSV G protein had clear colocalization. To further detect whether early endosomes and late endosomes play a role in the entry of BRSV into MDBK cells, we silenced Rab5 in MDBK cells with the siRNA of Rab5 (Figure 6A), and then inoculated BRSV for 24 h to detect viral replication. The results of virus titer and virus showed that silencing Rab5 significantly inhibited BRSV replication (Figures 6B,C). After silencing Rab5 of MDBK cells, BRSV was inoculated for 1 h, and BRSV entry was detected by qPCR, virus copies detection showed that BRSV entry was significantly inhibited (Figure 6D). To further investigate the involvement of Rab5 in BRSV entry, we examined the co-localization of Rab5 and BRSV G protein at 30 min after BRSV infected MDBK cells. The result of laser confocal showed clear colocalization of Rab5 and G protein (Figure 6E). Subsequently, we silenced Rab7 in MDBK cells by Rab7 siRNA (Figure 6F), and then inoculated BRSV for 24 h to detect viral replication. The results of virus titer and virus copies indicated that silencing Rab7 inhibited BRSV replication (Figures 6G,H), virus copies detection showed that BRSV entry was significantly inhibited (Figure 6I). Furthermore, we detected the co-localization of Rab7 and BRSV G protein at 60 min after BRSV infected MDBK cells, the result of laser confocal showed that Rab7 and G protein had obvious colocalization (Figure 6J). These results indicated that entered BRSV is trafficked to late endosomes via early endosomes.

### 3.7 PI3K-Akt and Src-JNK signaling pathways regulate CHC and dynamin-2 to promote BRSV entry

**Figure 7:**
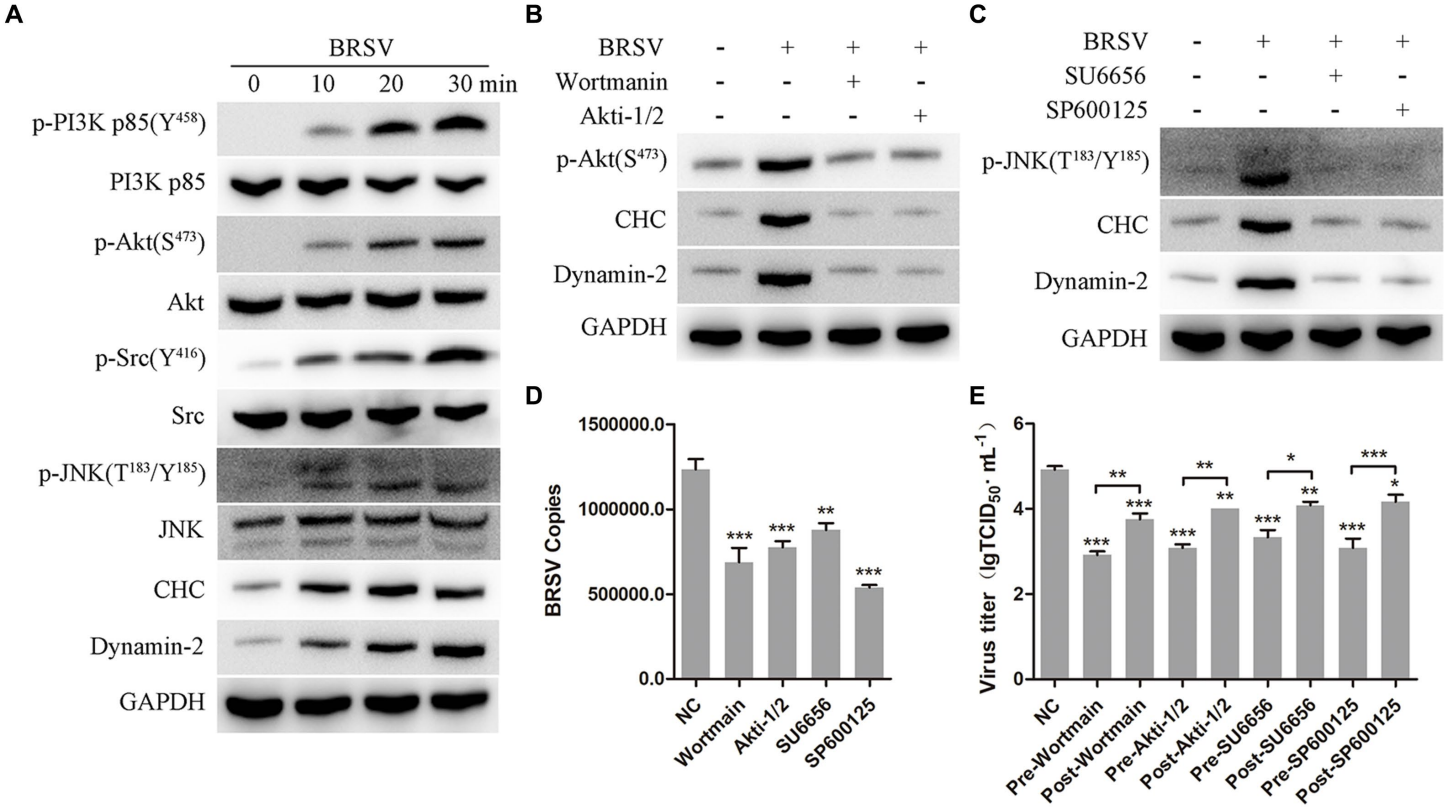
PI3K-Akt and Src-JNK signaling pathways regulate the expression of CHC and dynamin-2. **(A)** MDBK cells were infected with BRSV at an MOI of 2 for 0, 10, 20 or 30 min to detect designated proteins by western blot. **(B)** MDBK cells were pretreated with wortmannin (2.5 μM) or Akti-1/2 (10 μM), infected with BRSV at an MOI of 2 for 30 min, and designated proteins were detected by western blot. **(C)** MDBK cells were pretreated with SU6656 (2 μM) and SP600125 (8 μM), infected with BRSV for 30 min, and designated proteins were detected by western blot. **(D)** MDBK cells were pretreated with wortmannin, Akti-1/2, SU6656 or SP600125 for 1 h, bound with BRSV at an MOI of 2, transferred to 4°C for 1 h, and then transferred to 37°C for 1 h. The infected cells were collected, BRSV entry was analyzed by virus copies. **(E)** Wortmannin, Akti-1/2, SU6656 or SP600125 was added 1 h before or after BRSV infecting MDBK cells, the cells bound with BRSV at 4°C for 1 h were transferred to 37°C for 24 h. BRSV entry and replication were analyzed by virus titer. ^*^p < 0.05; ^**^p < 0.01; ^***^p < 0.001.

It has been reported that the PI3K-Akt and Src-JNK signaling pathways can regulate clathrin-mediated endocytosis and promote virus entry into cells, such as HSV-1, EBoV and BEFV (Li et al., 2010; Feng et al., 2011; Cheshenko et al., 2013; Cheng et al., 2015). We aimed to investigate the involvement of the PI3K-Akt and Src-JNK signaling pathways in BRSV entry into MDBK cells. The expression levels of p-PI3K, PI3K, p-Akt, Akt, p-Src, Src, p-JNK, JNK, CHC, and dynamin-2 were examined during the early stage of BRSV infection. The detection results showed that the expression of p-PI3K, p-Akt, p-Src, p-JNK, CHC and dynamin-2 gradually increased in the early stage of BRSV infecting MDBK cells (Figure 7A). To assess the impact of the PI3K-Akt and Src-JNK signaling pathways on clathrin-mediated endocytosis, MDBK cells were treated with wortmannin (2.5 μM, a PI3K inhibitor) or Akti-1/2 (10 μM, an Akt inhibitor) and then infected with BRSV for 30 min. The expression of p-Akt, CHC, and dynamin-2 was found to be promoted by BRSV infection, but their overexpression was reduced by wortmannin and Akti-1/2 (Figure 7B). Similarly, MDBK cells were treated with SU6656 (2 μM, a Src inhibitor) and SP600125 (8 μM, a JNK inhibitor) and then infected with BRSV for 30 min, the result showed the overexpression of p-JNK, CHC and dynamin-2 were reduced by wortmannin and Akti-1/2 (Figure 7C). Next, we examined the roles of PI3K-Akt and Src-JNK signaling pathways in BRSV entry. MDBK cells pretreated at 37°C with wortmannin, Akti-1/2, SU6656 or SP600125 for 1 h were incubated with BRSV at 4°C for 1 h and transferred to 37°C for 1 h, the result of virus copies showed that wortmannin, Akti-1/2, SU6656 or SP600125 inhibited BRSV entry into MDBK cells (Figure 7D). We added wortmannin, Akti-1/2, SU6656 or SP600125 to MDBK cells 1 h before and after BRSV inoculation to examine the roles of PI3K-Akt and Src-JNK signaling pathways in BRSV entry and replication, the cells inoculated with BRSV only were used as controls. Virus titers suggested that these inhibitors blocked BRSV entry and replication (Figure 7E). These results demonstrate that the PI3K-Akt and Src-JNK signaling pathways regulate clathrin-mediated endocytosis to promote BRSV entry into MDBK cells.

## 4 Discussion

In the process of virus infection, virus must enter the cytoplasm from outside the cell to complete its own replication within the cell (Yamauchi and Helenius, 2013). Therefore, understanding the manner and mechanism of virus entry into the cell is of great significance in preventing virus infection. Endocytosis is a common method by which viruses enter cells (Mercer et al., 2010). BRSV is an RNA virus with envelope, belongs to the paramyxovirus family. It causes acute, febrile, and highly infectious respiratory diseases in cattle (Larsen, 2000), resulting in significant economic losses to the cattle industry (Santos-Rivera et al., 2022). Previous reports have shown that various enveloped RNA viruses, such as Severe acute respiratory syndrome (SARS), Zika virus (ZIKV), PEDV, and Bovine parainfluenza virus 3 (BPIV3), can enter cells through endocytosis (Inoue et al., 2007; Li et al., 2020; Wei et al., 2020; Pan et al., 2021). In addition, a variety of DNA viruses can also enter host cells by this way, such as HSV-1, Bovine herpesvirus-1 (BoHV-1) and Feline Herpesvirus type 1 (FeHV-1; Liu and Cohen, 2015; Ferrara et al., 2023; Liu et al., 2023). However, the manner and mechanism of Respiratory syncytial virus (RSV) entry into cells have not been reported in detail. In this study, we explored the manner and mechanism of BRSV entering MDBK cells. Our findings demonstrate that BRSV can enter MDBK cells through clathrin-mediated endocytosis rather than caveolin-mediated endocytosis and macropinocytosis. The entered BRSV is localized in early endosomes and late endosomes in sequence. The process of BRSV entry through clathrin-mediated endocytosis is regulated by PI3K-Akt and Src-JNK signaling pathways.

The low pH environment of the endosome plays a crucial role in endocytosis and facilitates the transport of viruses into the cytoplasm (Pelkmans and Helenius, 2003; Marsh and Helenius, 2006). Previous studies have demonstrated that paramyxoviruses like Newcastle disease virus (NDV) and BPIV3 require a low pH environment for cell entry (Zhao et al., 2021). To investigate the impact of the endosomal acidic environment on BRSV entry, two inhibitors of endosomal acidification, NH4Cl and bafilomycin A1, were utilized. The results of virus titer, virus copies and IFA showed that the low pH environment of the endosome was necessary for BRSV to enter MDBK cells. Bafilomycin A1 has been shown to reduce virus titer during FeHV-1 infecting permissive cells in previous studies (Ferrara et al., 2023). Additionally, both NH_4_Cl and bafilomycin A1 have demonstrated inhibitory effects on BoHV-1 entry, aligning with our findings (Liu et al., 2023).

Dynamin, a GTPase, plays an important role in the process of cell endocytosis, acting like ‘scissors’ to separate the invaginated vesicle from the cell membrane and facilitate efficient virus transport into the cytoplasm (De Camilli et al., 1995; Liu and Robinson, 1995; Ramachandran and Schmid, 2018). It is a significant regulator of clathrin-mediated endocytosis and caveolin-mediated endocytosis (Orth et al., 2002; Sun and Tien, 2013). In order to detect the specific manner of BRSV entering MDBK cells, we tested whether dynamin played a role in this process. The results of virus titer, virus copies and IFA showed that dynamin was necessary for BRSV entering cells. Dynamin-2, an important member of dynamin family, was also involved in BRSV entry, and co-locate with BRSV G protein in the early stage of BRSV infection. These results show that dynamin is involved in BRSV entry, and suggest that BRSV may enter cells through clathrin-mediated endocytosis or caveolin-mediated endocytosis.

Clathrin-mediated endocytosis is a common way by which viruses enter cells, relying on dynamin. Previous studies have reported that BPIV3, a paramyxovirus, can enter MDBK cells through clathrin-mediated endocytosis. Chlorpromazine, an inhibitor of the clathrin-dependent endocytosis, can effectively inhibit the replication or entry of a variety of viruses, such as Hepatitis B virus (HBV), SARS and BPIV3 (Inoue et al., 2007; Herrscher et al., 2020; Pan et al., 2021). In our study, we demonstrated that BRSV enters MDBK cells via clathrin-mediated endocytosis by adding inhibitor chlorpromazine. Furthermore, we also reveal that CHC, a crucial functional domain of clathrin, is involved in the process of BRSV entry and exhibits clear colocalization with the BRSV G protein during the early stages of entry into MDBK cells. These results suggest that BRSV can enter MDBK cells through clathrin-mediated endocytosis.

Caveolin-mediated endocytosis, along with clathrin-mediated endocytosis, is a common manner for virus entry which also relies on dynamin. It has been reported that NDV, a paramyxovirus, can enter host cells through caveolin-mediated endocytosis. The uptake capacity of caveolae is necessary for the functioning of caveolin-mediated endocytosis (Shikanai et al., 2018), and cholesterol is essential for caveolae formation (Xia et al., 2018). To investigate the role of caveolin-mediated endocytosis in BRSV entry, we employed two cholesterol-consuming reagents, nystatin and MβCD. The results showed that neither nystatin nor MβCD had any effect on BRSV entry. Additionally, caveolin-1, an important member of caveolin family, did not affect BRSV entry. These results suggest that caveolin-mediated endocytosis does not play a role in BRSV entry into MDBK cells.

In addition to clathrin-mediated endocytosis and caveolin-mediated endocytosis, macropinocytosis is a common route for viral entry into cells (Coyne et al., 2007; Krieger et al., 2013). During the early stage of virus infection, virus binds to cell receptor and activates a variety of signaling pathways within the cell, which leads to actin rearrangement and the formation of irregular ruffles. The irregular ruffles envelop the virus-receptor complex, forming macropinosomes that transport the virus-receptor complex into cells (Mercer et al., 2010; Han et al., 2016). The formation of macropinosomes depends on NHE (Swanson and Yoshida, 2019; Salloum et al., 2023). EIPA, an inhibitor of NHE (Koivusalo et al., 2010), and IPA-3, an inhibitor that indirectly blocks actin rearrangement (Han et al., 2016), were used to investigate the role of macropinocytosis in BRSV entry. The results of virus titer and virus copies proved that EIPA and IPA-3 did not affect BRSV entry. These results indicate that BRSV entry is independent on macropinocytosis.

Virus can hijack Rab GTPases to promote their entry into MDBK cells through endocytosis (Rink et al., 2005; Mercer et al., 2010; Su and Zheng, 2021). Upon entering the cell through endocytosis, the virus initially localizes in the early endosome which is rich in Rab5. The early endosome gradually matures into the late endosome (Hanson and Cashikar, 2012), where Rab5 is released and Rab7 becomes associated with the late endosome during this maturation process (Rink et al., 2005; Wei et al., 2020). Therefore, we selected Rab5 and Rab7 as the signature proteins of early endosome and late endosome to investigate the role of endosome in BRSV entry. The results of viral titer and viral copies revealed that silencing Rab5 and Rab7 in MDBK cells could significantly inhibit the replication and entry of BRSV, and colocalization of Rab5/Rab7 and BRSV G protein was prominently observed at various time points post BRSV infection in MDBK cells. These results suggest that Rab protein plays an important role in the process of BRSV entry, entered BRSV is trafficked to late endosome via early endosomes.

Previous studies have shown that PI3K-Akt signaling pathway can regulate virus replication or entry, such as HSV-1, FeHV-1, BEFV and EBoV (Li et al., 2010; Feng et al., 2011; Cheshenko et al., 2013; Cheng et al., 2015; Liu and Cohen, 2015; Ferrara et al., 2023). Additionally, the activation of the Src-JNK signaling pathway has been observed during BEFV infection, promoting clathrin-dependent endocytosis and facilitating BEFV entry into cells (Cheng et al., 2015). In this study, our results also indicate that PI3K-Akt and Src-JNK signaling pathways can control the expression of CHC and dynamin-2 to regulated BRSV entry.

## 5 Conclusion

**Figure 8:**
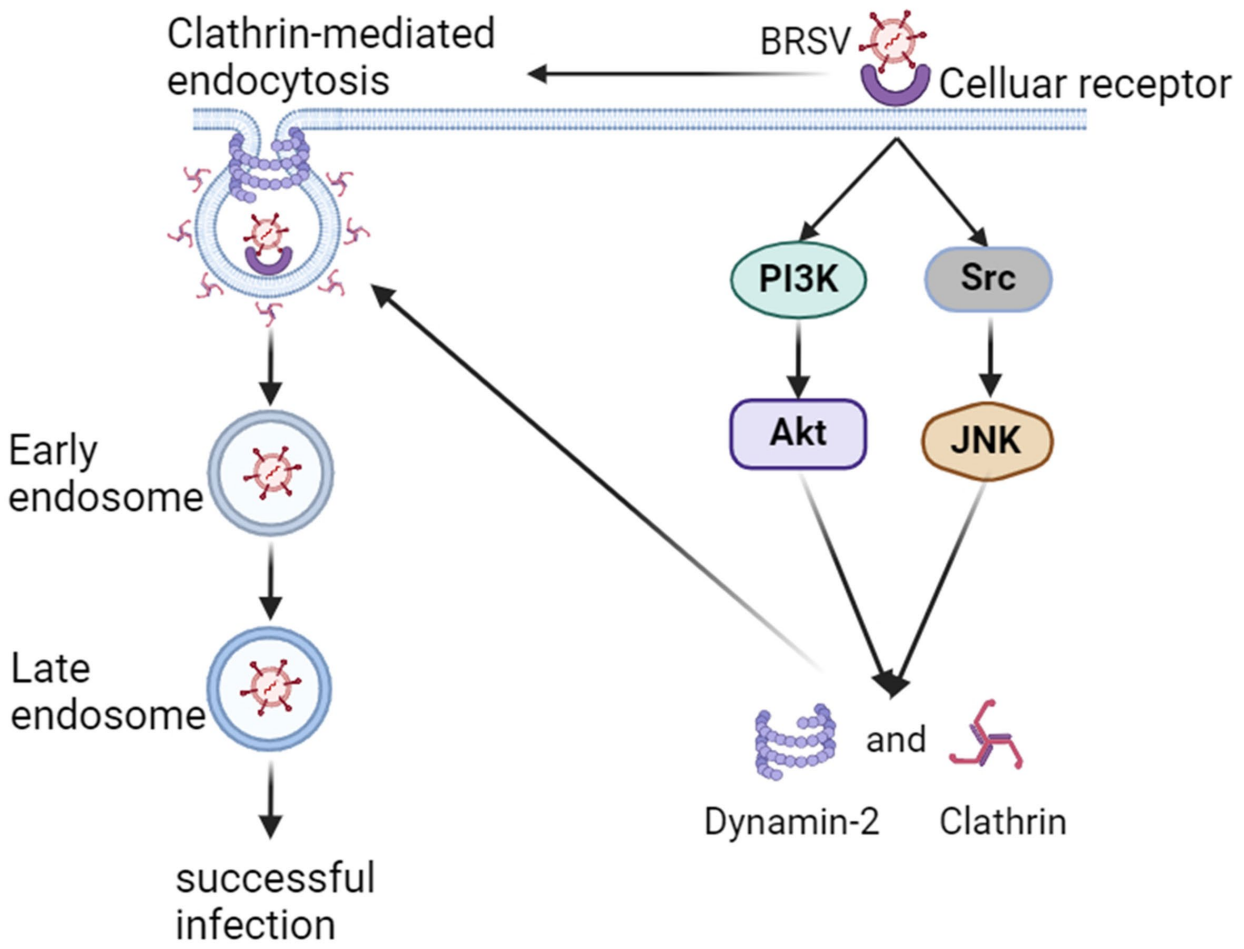
Model of BRSV entry into MDBK cells. In the early stage of BRSV infection, BRSV binding cellular receptor activates PI3K-Akt and Src-JNK signaling pathways to enhance the expression of CHC and dynamin-2, further promotes BRSV entry into the cells through clathrin-mediated endocytosis. The internalized BRSV is traced to is trafficked to late endosome via early endosome.

In summary, our findings indicate that BRSV can enter MDBK cells through endocytosis, specific endocytosis pathway is clathrin-mediated endocytosis rather than caveolin-mediated endocytosis and macropinocytosis, and the entered BRSV is located in the early endosome and late endosome successively. The process of BRSV entry is regulated by PI3K-Akt and Src-JNK signaling pathways (Figure 8).

## Data availability statement

The original contributions presented in the study are included in the article/supplementary material, further inquiries can be directed to the corresponding authors.

## Author contributions

YL: Investigation, Methodology, Writing – original draft. DY: Investigation, Writing – original draft. WJ: Writing – review & editing. TC: Writing – review & editing. JK: Writing – review & editing. ZW: Conceptualization, Data curation, Methodology, Resources, Supervision, Writing – review & editing. FW: Conceptualization, Data curation, Methodology, Resources, Supervision, Writing – review & editing.
